# Body mass index has a curvilinear relationship with the percentage of body fat among children

**DOI:** 10.1186/1756-0500-4-301

**Published:** 2011-08-18

**Authors:** Bruno Federico, Filomena D'Aliesio, Fabio Pane, Giovanni Capelli, Angelo Rodio

**Affiliations:** 1 Department of Health and Sport Sciences, University of Cassino, Cassino, Italy

## Abstract

**Background:**

Body Mass Index (BMI), which is defined as the ratio between weight (in kg) and height (in m^2^), is often used in clinical practice as well as in large scale epidemiological studies to classify subjects as underweight, normal weight, overweight or obese. Although BMI does not directly measure the percentage of Body Fat (BF%), it is widely applied because it is strongly related with BF%, it is easy to measure and it is an important predictor of mortality. Among children, age and sex-specific reference values of BMI, known as percentiles, are used. However, it is not clear how strong the relationship between BMI and BF% is among children and whether the association is linear. We performed a cross-sectional study aiming at evaluating the strength and shape of the relationship between BMI and BF% among school-aged children aged 6-12 years.

**Findings:**

The study was conducted on a sample of 361 football-playing male children aged 6 to 12 years in Rome, Italy. Age, weight, height and skinfold thickness were collected. BF% was estimated using 4 skinfold equations whereas BMI was converted into BMI-for-age z-score. The relationship between these variables was examined using linear regression analyses. Mean BMI was 18.2 (± 2.8), whereas BF% was influenced by the skinfold equation used, with mean values ranging from 15.6% to 23.0%. A curvilinear relationship between BMI-for-age zscore and BF % was found, with the regression line being convex. The association between BMI-for-age zscore and BF% was stronger among overweight/obese children than among normal/underweight children. This curvilinear pattern was evident in all 4 skinfold equations used.

**Conclusions:**

The association between BMI-for-age zscore and BF% is not linear among male children aged 6-12 years and it is stronger among overweight and obese subjects than among normal and underweight subjects. In this age group, BMI is a valid index of adiposity only among overweight and obese subjects.

## Findings

The percentage of Body Fat (BF%) is currently estimated using several methods, which include underwater weighing, air displacement plethysmography, dual-energy X-ray absorptiometry and bioelectrical impedance analysis [1]. Most of these methods are either extremely expensive or they require complex technologies and are therefore of limited use in clinical practice as well as in large scale epidemiological studies. Within these contexts, anthropometric methods may instead prove to be extremely useful [2]. Body Mass Index (BMI), defined as the ratio between body mass (in kg) and height (in m^2^), is commonly used to define the conditions of obesity and overweight among adults [3]. Although BMI does not directly measure BF%, it is a valid indicator of BF% [2] and it is very easy to measure. BMI is also an important predictor of mortality [4,5]. Among adults, BMI cut-off values of 25 and 30 define the conditions of overweight and obesity, respectively, whereas among children age and gender specific reference values of BMI, known as percentiles, are used [6]. Previous studies found that BMI was correlated with BF% among children, too [7,8]. However, all these studies assumed the existence of a linear association between these two variables. In this study, we aimed at evaluating the strength and shape of the relationship between BMI and BF% among school-aged children.

Using a cross-sectional design, we examined a sample of 361 football-playing male children aged 6 to 12 years enrolled in the child division of Lazio football team in Rome, Italy. The study protocol was approved by the ethical review board of the Lazio football team child division. Informed consent to the study was given by either of the parents. Data collection was carried out by ad-hoc trained sports medicine physicians and anthropometric measurements such as weight, height and skinfolds thickness were performed for each subject. Biceps, triceps, subscapular, supra-iliac and calf skinfolds were all measured with Harpenden skinfold calipers. Skinfold thickness measurement was performed three times on the not dominant limb by a single operator, and the mean value of the three measurements was used. Data were first registered on a paper form and then stored into an electronic format using the software Epidata Entry. Data entry was performed in duplicate to minimize entry errors.

We used 4 skinfold equations in order to estimate BF % of each subject [9]. These equations, developed by Deurenberg, Dezenberg and Slaughter, are based on demographic data (age and gender), weight and different skinfolds depending on the equation used. BMI data were transformed into BMI-for-age z-scores using the LMS method and the reference data available from the 2000 CDC Growth Reference in the US [10,11]. Linear regression analyses were performed to evaluate the relationship between BMI z-score (the independent variable) and BF% (the dependent variable). Subjects were also classified as overweight or obese according to the BMI cut-off points developed in an international survey [6]. All statistical analyses were carried out with Stata 11.2.

Sample descriptive statistics (mean and standard deviation) are shown in Table 1. Mean BMI was 18.2 (± 2.8), whereas BF% was influenced by the skinfold equation used, with mean values ranging from 15.6% using Slaughter 1 equation to 23.0% using Dezenberg equation. About 1 in 3 children was either overweight (24.4.%) or obese (5.6%).

**Table 1:** Descriptive statistics of the sample

	N	Mean	SD
**Height (cm)**	359	139.1	10.1
**Weight (Kg)**	361	35.9	9.1
**Body mass index (Kg/m^2^)**	359	18.2	2.8
**Skinfold thickness (mm)**			
Biceps	359	7.4	3.9
Triceps	360	11.5	4.8
Subscapular	359	8.9	5.5
Suprailiac	359	8.4	5.8
Calf	354	8.4	4.4
**Percentage body fat**			
Deurenberg equation	357	17.9	5.2
Dezenberg equation	360	23.0	5.3
Slaughter equation 1	354	15.6	6.4
Slaughter equation 2	359	18.5	7.0

**Figure 1:**
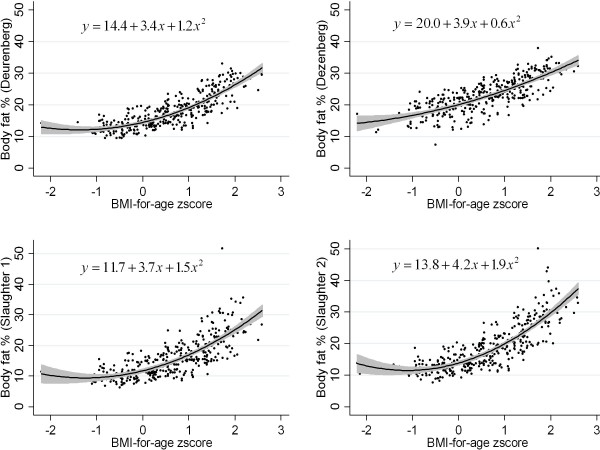
**Scatterplot and regression line (with 95% Confidence Intervals) of Body Fat percentage versus BMI-for-age zscore according to the four skinfold equations used**.

A curvilinear relationship between BMI-for-age zscore and BF% was found, with the regression line being convex (Figure 1). This curvilinear pattern is evident in each of the 4 skinfold equations that we used to predict BF%. The addition of a quadratic term to the linear regression model significantly increased the proportion of the variability in BF% explained by the model: in the case of Deurenberg equation (top left panel in Figure 1), R^2 ^increased from 0.66 to 0.72. Values for R^2 ^for the quadratic model were 0.63 (Dezenberg), 0.60 (Slaughter 1), and 0.69 (Slaughter 2).

When the regression analysis was performed separately among normal and overweight/obese subjects, beta coefficients (which indicate the slope of the regression lines) were significantly smaller for the normal weight category than in the overweight/obese category. In the case of Deurenberg equation, beta coefficient was 2.9 among the former category, and 6.7 among the latter category (p < 0.001). Differences of a similar extent between beta coefficients were observed for the other 3 skinfold equations.

To conclude, our study shows that there is a curvilinear relationship between BMI-for-age z-score and BF%, with strong correlations existing only among overweight and obese subjects. This finding is confirmed by the results of other studies, which used different methods of analyses [12-14], and it implies that the use of BMI as an index of adiposity among children is valid only among overweight and obese subjects.

## List of abbreviations used

BMI: Body Mass Index; BF%: Body Fat percentage

## Competing interests

The authors declare that they have no competing interests.

## Authors' contributions

BF designed the study and drafted the manuscript. FDA and FP contributed to data collection and data analyses and they both contributed to revising the drafts. GC participated in the design of the study and performed data analyses. AR had the original idea of the study, he was responsible for data collection and he revised the drafts. All authors read and approved the final manuscript.
